# Identification of B-Cell Epitopes of HspA from *Helicobacter pylori* and Detection of Epitope Antibody Profiles in Naturally Infected Persons

**DOI:** 10.3390/vaccines10010065

**Published:** 2021-12-31

**Authors:** Xin Zhang, Shuli Sang, Qing Guan, Haoxia Tao, Yanchun Wang, Chunjie Liu

**Affiliations:** 1 State Key Laboratory of Pathogen and Biosecurity, Institute of Biotechnology, Academy of Military Medical Sciences, 20 Dongda Street, Fengtai District, Beijng 100071, China; zhangxin8485@126.com (X.Z.); sslsandra@126.com (S.S.); yiyiyiyan610@163.com (Q.G.); taohaoxia@126.com (H.T.); 2 Department of Pharmacy, Medical Supplies Center, Chinese PLA General Hospital, 28 Fuxing Road, Haidian District, Beijing 100853, China

**Keywords:** *Helicobacter pylori*, B-cell epitope, heat-shock protein A, epitope antibody profiles

## Abstract

*Helicobacter pylori* (*H. pylori*), heat-shock protein A (HspA), is a bacterial heat-shock chaperone that serves as a nickel ion scavenging protein. Ni^2+^ is an important co-factor required for the maturation and enzymatic activity of *H. pylori* urease and [NiFe] hydrogenase, both of which are key virulence factors for pathogen survival and colonization. HspA is an important target molecule for the diagnosis, treatment, and immune prevention of *H. pylori*. In this work, HspA was truncated into five fragments to determine the location of an antigen immunodominant peptide. A series of overlapping, truncated 11-amino-acid peptides in immunodominant peptide fragments were synthesized chemically and screened by ELISA. The immunogenicity and antigenicity of the screened epitope peptides were verified by ELISA, Western blot, and lymphocyte proliferation tests. Two novel B-cell epitopes were identified, covering amino acids 2–31 of HspA, which are HP11 (2–12; KFQPLGERVLV) and HP19 (18–28; ENKTSSGIIIP). The antiserum obtained from HP11-KLH and HP19-KLH immunized mice can bind to naive HspA in *H. pylori* SS2000, rHspA expressed in *E. coli*, and the corresponding GST fusion peptide. Among HspA seropositive persons, the seropositive rates of HP11 and HP19 were 21.4% and 33.3%, respectively. Both of the B-cell epitopes of HspA are highly conserved epitopes with good antigenicity and immunogenicity.

## 1. Introduction

*Helicobacter pylori* (*H. pylori*), a spiral-shaped, microaerophilic, Gram-negative bacterium that colonizes the stomach of nearly half of the world’s population, has been classified as a group I carcinogen by the International Agency for Research on Cancer (IARC). *H. pylori* is highly associated with chronic active gastritis, peptic ulcers, lymphoma of the mucosa-associated lymphoid tissue (MALT), and gastric adenocarcinoma [1,2,3]. Furthermore, *H. pylori* infection is usually acquired in childhood and, if it is not cured, can cause lifelong infection [4]. The current therapeutic approach is based on a triple or quadruple combination of antibiotics, proton pump inhibitors, and bismuth compounds, as one course of treatment lasting 10–14 days [5]. However, the eradication rate of *H. pylori* has a downward trend in the world, along with a rapid increase in clarithromycin resistance in many countries during the past decade [6]. In addition to increasing reports of antibiotic-resistant strains, high treatment costs, poor drug compliance, and a high rate of infection recurrence are the main reasons for the failure of large-scale control of *H. pylori* infection. Therefore, research on immune response and immune protection mechanisms against *H. pylori* are of positive significance for overcoming the problems of current treatment methods and, for more effective evaluation, prevention and treatment of *H. pylori* infection.

*H. pylori* heat-shock protein A (HspA) is a bacterial heat-shock chaperone with an essential function as an Ni-ion scavenging protein, which is one of the key virulence factors and protective antigens against *H. pylori* infection [7]. HspA consists of 118 amino acids divided into two domains: the A domain (1–90), which shares sequence similarity to the GroES sequence [8], and the B domain (91–118), which is unique to *H. pylori* and *Helicobacter acinonuchis*, and it contains eight histidines and four cysteines [9,10]. HspA is involved in intracellular nickel sequestration and detoxification, which is involved in maintaining Ni^2+^ homeostasis of *H. pylori*. Metallic nickel is an important co-factor for the survival and colonization of *H. pylori*, affecting the activity and function of urease and [NiFe] hydrogenase [10,11,12]. HspA is an important target molecule for the diagnosis, treatment, and immune prevention of *H. pylori*. HspA antibodies can be detected in about 40% of persons with *H. pylori* infection [13]. Bi^3+^ irreversibly binds to HspA and interferes with its biological functions. This action serves as the main mechanism of bismuth compound treatment effects [9,14]. HspA has an obvious immune protection effect against *H. pylori* in the mouse model [15,16].

It is well known that the body generates an adaptive immune response by recognizing the epitopes of pathogenic bacteria to resist the invasion of foreign pathogens. Therefore, the acquisition of epitopes is a very important stage in the diagnosis of diseases, understanding of immune protection mechanisms, and vaccine development. In this study, we identified two novel B-cell epitopes from HspA and detected the antibody expression profiles of these epitope peptides in an *H. pylori*-naturally-infected population. Our findings provide a basis for further study on the role of HspA in immune response and immune prevention caused by *H. pylori* infection.

## 2. Materials and Methods

### 2.1. Bacterial Strains and Culture Conditions

Two *H. pylori* strains, Sydney strain 2000 (SS2000) and NCTC11637, were cultured on Campylobacter Agar Base plates (CDRC, Shanghai, China) containing 7% fetal bovine serum, and cultivated for 3–4 days at 37 °C under microaerophilic conditions with 80% N_2_, 10% CO_2_, and 5% O_2_. Then, the colonies were scraped with phosphate-buffered saline (PBS), washed twice, and then centrifuged at 10,000× *g* at 4 °C for 10 min. The SS2000 pellets were resuspended in PBS, sonicated, and centrifuged for 20 min to obtain the supernatant. The supernatant, containing HspA, was used for Western blotting. NCTC11637 pellets were used for preparing genomic DNA.

### 2.2. Construction, Expression, and Purification of H. pylori Recombinant HspA (rHspA) and GST Fusion Peptides

The gene sequences of HspA were amplified from the genome of NCTC11637 by PCR and cloned into the expression vector pGEX-6P-1(+) plasmid (GE Healthcare, Pittsburgh, PA, USA), placed between *BamH*I and *Not*I restriction sites (Primer pair: 5′-CGCGGATCCAAGTTTCAACCATTAG-3′ and 5′-AAATATGCGGCCGCTTAGTGTTTTTTGTGATC-3′). The recombinant plasmid was transformed into *E. coli* BL21 (DE3) cells (Cwbio, Suzhou, China), and the protein expression was induced with 1 mM IPTG. The cells were collected by centrifugation, resuspended in PBS, and then disrupted by ultrasonication and centrifuged to remove insoluble cellular components. The recombinant GST-HspA fusion proteins were purified by Price Glutathione Superflow Agarose (Thermo, Rockford, IL, USA) according to the instructions. Then, the GST tag was excised with PreScission Protease (Beyotime, Nanjing, China) and removed using GST-tag purification resin (BeyoGold, Nanjing, China). The purity and concentration of the recombinant protein rHspA were determined by SDS-PAGE and BCA assay.

For GST fusion peptides expression, two complementary oligonucleotides GST-HP11F/R or GST-HP19F/R were annealed and formed the sticky ends of *BamH*I and *Xho*I restriction endonuclease (GST-HP11F: 5′-GATCCAAGTTTCAACCATTAGGAGAAAGGGTCTTAGTATAAC-3′; GST-HP11R: 5′-TCGAGTTATACTAAGACCCTTTCTCCTAATGGTTGAAACTTG-3′; GST-HP19F: 5′-GATCCGAGAACAAAACCAGTTCAGGCATTATCATCCCTTAAC-3′; GST-HP19F: 5′-TCGAGTTAAGGGATGATAATGCCTGAACTGGTTTTGTTCTCG-3′). They were cloned into the expression vector pGEX-6P-1(+) (GE Healthcare, USA) and placed between *BamH*I and *Xho*I restriction sites. The expression and purification of GST-HP11 and GST-HP19 were similar to rHspA.

### 2.3. Synthesis of Peptides and Keyhole Limpet Hemocyanin (KLH)-Conjugated Peptides

The overlapping peptides, which covered the full length of HspA and the immunodominant region of HspA, were synthesized by GeneScript (Nanjing, China) using the standard, solid-phase fluorenylmethyloxycarbonyl (Fmoc) method. The purity was estimated to be >95% using high-pressure liquid chromatography and identified by mass spectrometry. The peptides were dissolved in ultrapure water or DMSO at a concentration of 10 mg/mL and stored at −20 °C. The 118 amino acids of HspA were divided into five peptide segments containing 29–30 amino acids, and each of them overlapped by eight amino acids. In order to enhance the immunogenicity of the peptide, we attached a cysteine to the C-terminuses of HP11 and HP19, and then conjugated them to KLH using the m-maleimidobenzoyl-N-hydroxysuccinimide ester (MBS) method to form KLH-conjugated peptides.

### 2.4. Immunization of Mice and Sample Collection

Female, specific-pathogen-free (SPF) BALB/c mice, aged 6–8 weeks, were purchased from Vital River Laboratory (Beijing, China) and bred under SPF conditions. An emulsion was prepared by mixing 50 µg rHspA or KLH-conjugated peptide with complete Freund’s adjuvant (Sigma-Aldrich, St. Louis, MO, USA) in volume ratio of 1:1 and was used to subcutaneously immunize mice on day 0. Immunization was boosted with the same antigen mixed with incomplete Freund’s adjuvant (Sigma-Aldrich, St. Louis, MO, USA) on days 10 and 20. Control mice were immunized with PBS. Mice were sacrificed on day 30, sera samples were collected for ELISA and Western blot, and splenocytes were collected for lymphocyte proliferation analysis. All animal experiments were performed in accordance with the guidelines of the Animal Care and Use Committee of the Academy of Military Medical Sciences.

### 2.5. Acquisition and Testing of Human Serum

This study was approved by the Ethics Committee of the Fourth Medical Center of PLA General Hospital and informed consent was obtained from the patients. The serum of inpatients was collected and the *H. pylori* IgG ELISA kit (IBL, Hamburg, Germany) was used to detect whether antibodies against *H. pylori* were present. The experimental operation was performed according to the manufacturer’s instructions. When the cut-off index (COI) > 1.2, the serum was considered positive for *H. pylori*, and when the cut-off index (COI) < 0.8, the serum was considered negative for *H. pylori*. Eighty-five cases of *H. pylori* positive sera were selected for HspA antibody and epitope antibody detection by ELISA, and 17 cases of *H. pylori* antibody negative sera were used as a control. The positive judgment limit of HspA antibody and epitope antibody was an OD value > average OD value of negative control serum × 2.1.

### 2.6. ELISAs for Peptides and rHspA

The B-cell epitopes of HspA were identified by ELISA. Briefly, 96-well ELISA plates (Costar, Kennebunk, ME, USA) were pretreated with 150 µL 2.5% glutaraldehyde at 37 °C for one hour and then washed with water four times. Each peptide was diluted in 0.1 mM carbonate buffer (pH 9.6) and coated onto ELISA plates (30 μg/mL, 100 μL/well), incubated overnight at 37 °C. Recombinant protein HspA (2 μg/mL, 100 μL/well) was coated overnight at 4 °C and the ELISA plates did not require pretreatment. The plates were washed three times with PBST, and then blocked with 200 μL of blocking solution at 37 °C for two hours. PBST containing 5% skimmed milk was used for mice sera, and protein-free T20 (PBS) Blocking Buffer (Thermo, Rockford, IL, USA) was used for human sera. Next, rHspA immune sera, KLH-conjugated peptide immune sera, or human sera were appropriately diluted in PBST containing 1% skimmed milk and then added to the plates (100 μL/well) and incubated at 37 °C for one hour. Sera from PBS immune mice or persons with negative *H. pylori* infection were used as a negative control. After washing the wells three times with PBST, HRP-conjugated rabbit anti-mouse IgG (1:5000 dilution, Abcam, Cambridge, UK) or goat anti-human IgG (1:20,000 dilution, Abcam, Cambridge, UK) were added to the wells (100 μL/well). The plates were incubated at 37 °C for one hour and washed again. Then 3,3,5,5-tetramethylbenzene-biphenyl (TMB) was added at 100 μL/well, and the reaction was allowed to progress for 20 min at room temperature in the dark, and it was terminated with 100 μL of 1 M H_2_SO_4_. The absorbance at 450 nm (A450) was measured in a SpectraMax i3x (Molecular Devices, San Jose, CA, USA). All samples were analyzed in duplicate, and the means of the replicates were reported in the results.

### 2.7. SDS-PAGE and Western Blot Analysis

Western blot analysis was performed on the sera immunized by KLH-conjugated peptides or rHspA with supernatants of *H. pylori* SS2000 fragments, rHspA, and GST fusion protein. Supernatants of *H. pylori* fragments, rHspA, GST fusion protein, and GST were denatured and separated by 15% SDS-PAGE, transferred onto a polyvinylidene fluoride (PVDF) membrane (Merck Millipore, Mannheim, Germany), and then blocked with PBST containing 5% skimmed milk at 37 °C for one hour to prevent non-specific protein binding. The membranes were incubated with a 1:500 dilution of serum immunized with KLH-conjugated peptide or rHspA at 37 °C for one hour, washed with PBST three times, and then incubated with HRP-conjugated rabbit anti-mouse IgG (1:5000 dilution, Abcam, Cambridge, UK) at 37 °C for one hour. The HRP Western Blot Analysis Kit (Easybio, Beijing, China) was used to detect the binding reaction.

### 2.8. Lymphocyte Proliferation Responses

Spleens were pressed through a fine nylon mesh using syringe plungers to prepare single cell suspensions. Cells were seeded (4 × 10^5^ cells per well) in RPMI-1640 medium (Gibco, Grand Island, NE, USA) containing 10% FBS in the presence of 10 μg/mL for each candidate peptide or 0.625 μg/mL ConA for 72 h at 37 °C and 5% CO_2_. Under the same conditions, the cells in the presence of media were only regarded as non-antigen-stimulated cells. Mice immunized with PBS served as a negative control group. The cells were pulsed with 20 μL Cell Counting Kit-8 (CCK8) solution (Dojindo, Kumamoto Ken, Japan) per well for the last four hours of culture, and the A450 was measured in a SpectraMax i3x (Molecular Devices, San Jose, CA, USA). The results were expressed as stimulation indexes (SI): SI = OD value of stimulated cultures/OD value of non-stimulated cultures.

### 2.9. Subsection Statistical Analysis

All data were analyzed using GraphPad Prism 8.0.2 (GraphPad Software, San Diego, CA, USA), and they were represented as mean ± standard deviation (S.D.). Means were compared using a paired two-tailed *t*-test, and a value of *p* < 0.05 was considered statistically significant.

## 3. Results

### 3.1. Subsection Preliminary Screening of Antigen Immunodominant Peptide Fragment in HspA

**Figure 1:**
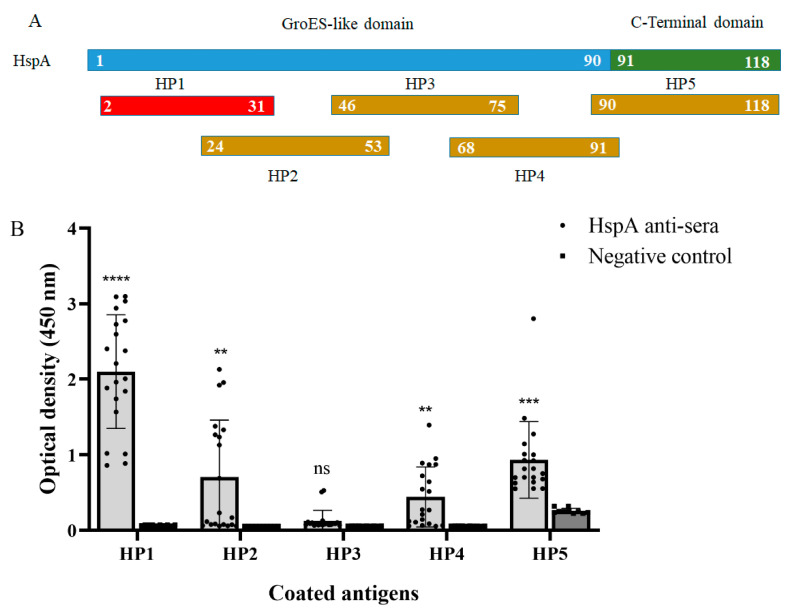
Preliminary screening of an antigen immunodominant peptide fragment in HspA. (**A**) The truncated fragment of HspA from *H. pylori* and its relative position. The antigen immunodominant region are marked in red. (**B**) The OD values of five fragments detected by ELISA with 20 mouse anti-rHspA sera (1:500 dilution). The data were presented as mean ± SD. ** *p* < 0.01, *** *p* < 0.001, **** *p* < 0.0001 vs. negative control.

Firstly, HspA was cut into five segments to primarily determine the location of antigen immunodominant peptide fragment as showed in Figure 1A. It was found that the originally designed fourth peptide (amino acid 68–97) was difficult to chemically synthesize due to serious hydrophobicity, and finally it was shortened to 24 amino acids (HP4, amino acid 68–91). Then, these five peptides were chemically synthesized and detected with 20 mouse anti-rHspA sera by ELISA. The results showed that HP1 could react with all anti-sera and the immune response was the strongest. Therefore, we inferred that HP1 was the antigen immunodominant region in HspA (Figure 1B).

### 3.2. Fine Localization of the Epitope in HP1

**Figure 2:**
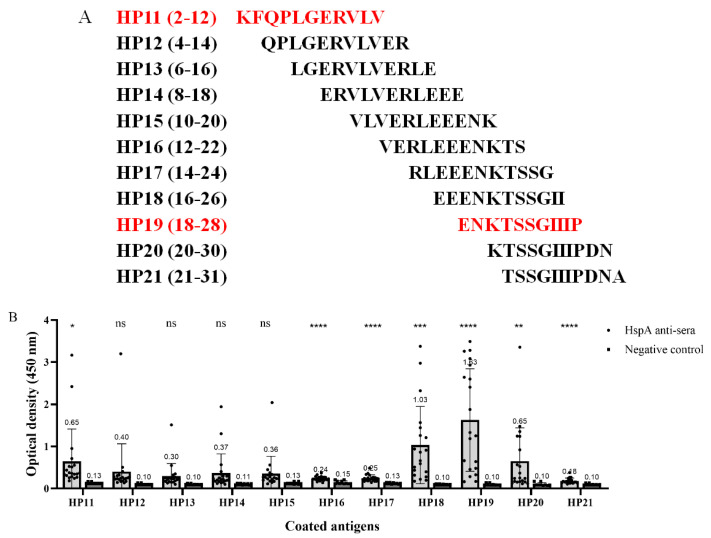
Fine localization of antigen epitopes in HP1. (**A**) Overlapping synthetic peptides covering HP1. Each peptide contains 11 amino acids with nine amino acids overlapping with the adjacent peptides. The red text shows the peptides with strong immune responses that may be the epitopes, according to the results of (**B**). (**B**) The OD value of 11 peptides detected by ELISA with mouse anti-rHspA sera (1:500 dilution). The data were presented as mean ± SD. * *p* < 0.05, ** *p* < 0.01, *** *p* < 0.001, **** *p* < 0.0001 vs. negative control.

For the fine mapping of the epitope of the HspA HP1 fragment, a set of 11 overlapped amino acid peptides covering HspA from 2 to 31 amino acids were synthesized and a total of 11 peptides were obtained (Figure 2A). The synthetic peptides were used as capture antigens in ELISA with the intensity of 11 peptides with 20 mouse anti-rHspA sera being detected. The test results showed that although HP11, HP16, HP17, HP18, HP19, HP20, and HP21 were significantly, or very significantly, different from the negative control, but only the ratio of HP11, HP18, HP19, and HP20 to the negative control group reached more than 2.1 times, and the immune response was the strongest (Figure 2B). The sequences of the three peptides HP18, HP19, and HP20 were similar. We believed that the epitope may have been located in HP19 (ENKTSSGIIIP), which had the strongest immune response. In addition, the immune response of HP11 (KFQPLGERVLV) was comparable to that of HP20, which may have been another epitope.

### 3.3. Detection of Immunogenicity and Immunoreactivity of Epitope HP11 and HP19

**Figure 3:**
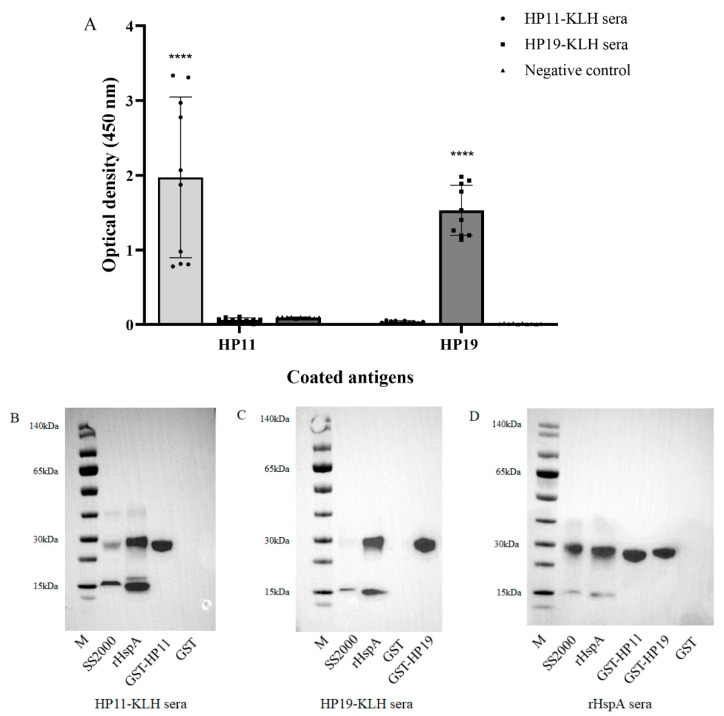
The immunogenicity and immunoreactivity of epitopes HP11 and HP19. (**A**) Reactivity of anti-KLH-conjugated peptide sera with synthetic peptides by ELISA. Synthetic peptides HP11 and HP19 as the coating antigen, KLH-conjugated peptide sera antibody (1:100 dilution) from mice as the primary antibody, and PBS immunized mouse sera as the negative control. The data were presented as mean ± SD. **** *p* < 0.0001 vs. negative control. (**B**) Western blotting analysis of anti-sera obtained by immunization with HP11-KLH. M: marker; lane 1: supernatant of ultrasonic broken material of *H. pylori* strain SS2000; lane 2: rHspA; lane 3: GST-HP11; lane 4: GST (negative control). (**C**) Western blotting analysis of anti-sera obtained by immunization with HP19-KLH. M: marker; lane 1: supernatant of SS2000; lane 2: rHspA; lane 3: GST (negative control); lane 4: GST-HP19. (**D**) Western blotting analysis of anti-sera immunized by rHspA with GST-HP11 and GST-HP19. M: marker; lane 1: supernatant of SS2000; lane 2: rHspA; lane 3: GST-HP11; lane 4: GST-HP19; lane 5: GST (negative control).

After initially identifying the two B-cell epitopes in HspA, we evaluated whether these epitopes could induce immune responses in mice. Six-to-eight-week-old female SPF BALB/c mice were injected subcutaneously with HP11 and HP19 KLH-conjugated peptides (called HP11-KLH and HP19-KLH) or PBS. ELISA was used to evaluate whether the immunization produced peptide-specific antibodies. The results showed that injection of the KLH-conjugated peptide could induce a strong immune response in mice, and the peptide-specific antibody titer was more than 1:1000. HP11 and HP19 were coated to react with all sera of mice immunized with KLH-conjugated peptides. The immune response generated by the self-peptide could be detected, and demonstrated that the peptide did not react with other KLH-conjugated peptides or PBS antiserum (Figure 3A).

In addition, Western blotting was performed to further evaluate anti-sera from immunized mice with a KLH-conjugated peptide. The *H. pylori* strain SS2000 and two GST fusion proteins, which contained epitope sequences named GST-HP11 and GST-HP19, were prepared for this assay. The rHspA protein was used as a positive control and GST protein was used as negative control. The results showed that the anti-sera from immunized mice with HP11-KLH and HP19-KLH could respond to naive HspA of *H. pylori* strains, the rHspA expressed in *E. coli*, and their own GST fusion protein but failed to react with the GST protein (Figure 3B,C). The results indicated that the KLH-conjugated peptide of two epitopes could evoke a humoral immune response specific to epitopes. Then, we used anti-sera immunized by rHspA to react with naive HspA from SS2000, rHspA protein, and the two GST fusion proteins GST-HP11 and GST-HP19 to verify the immunoreactivity of the GST fusion protein. Our findings confirmed that the two fusion proteins could react with the HspA whole protein anti-sera (Figure 3D).

### 3.4. Lymphocyte Proliferation Responses Test

**Figure 4:**
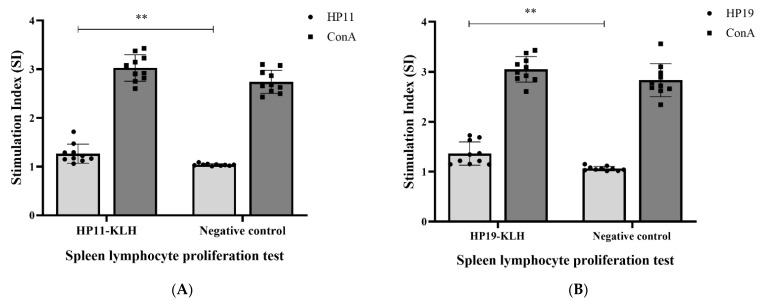
Antigen-specific lymphocyte proliferation responses. Epitope peptide stimulated the proliferation of spleen cells in HP11-KLH (**A**) and HP19-KLH (**B**) immunized mice. The same peptide stimulated PBS-immunized mice as a negative control, and ConA stimulation served as a positive control. Stimulation index (SI) = OD value of stimulated cultures/OD value of non-stimulated cultures. The data were presented as mean ± SD. ** *p* < 0.01 vs. negative control.

To further understand the immune responses induced by the epitopes, we examined the epitope-specific lymphocyte proliferative activity of spleen lymphocytes from mice immunized by the KLH-conjugated peptide. Overall, spleen lymphocyte stimulation indices were significantly higher in the KLH-conjugated peptide immunized groups than in the negative control (*p* < 0.01, Figure 4).

### 3.5. Amino Acid Homology and Human Serum Antibody Profile Analysis of Identified Epitopes

The basic partial alignment search tool (protein–protein BLAST) suite from the National Center for Biotechnology Information (NCBI) was used to analyze the amino acid sequence conservation of two epitopes. Three hundred and forty heat-shock proteins or co-chaperone GroESs from different *H. pylori* strains contained HP11 sequences. Among them, 334 (98.24%) had 100% sequence coverage and sequence homology with HP11 epitope peptide (Supplementary Table S1). There were 353 heat-shock proteins or co-chaperone GroESs from different *H. pylori* strains containing HP19 sequences, of which 345 (97.73%) had 100% sequence coverage and 100% homology with HP19 epitope peptide (Supplementary Table S2).

**Figure 5:**
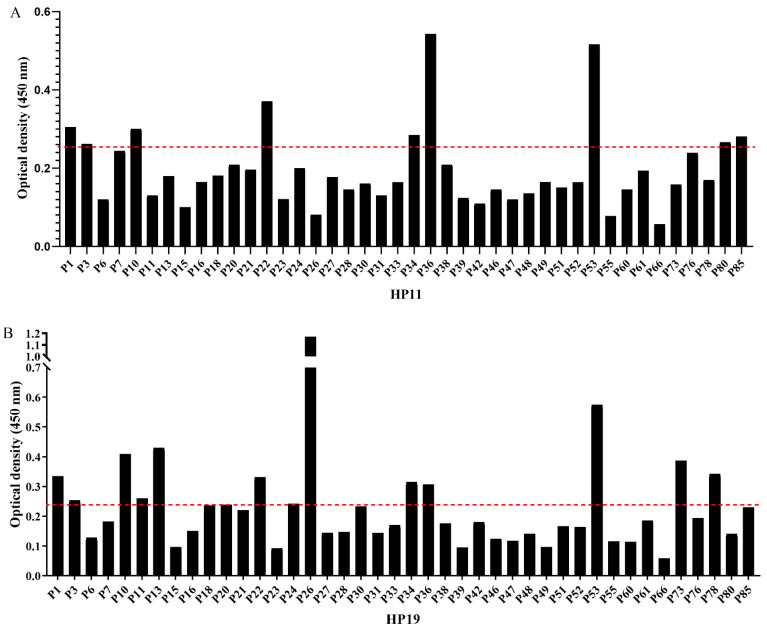
Human serum antibody profile of epitope HP11 (**A**) and HP19 (**B**). The OD values of HP11 and HP19 detected by ELISA with 42 anti-HspA seropositive samples (1:100 dilution). Seventeen *H. pylori* antibody negative and HspA antibody negative sera were used as negative controls. Positive standard: OD value > average OD value of negative control sera × 2.1 (the red line).

We next examined the antibody profile of the identified B-cell epitopes in HspA with sera from *H. pylori* IgG-positive persons. Serum samples of 85 *H. pylori* IgG-positive persons confirmed by *H. pylori* IgG ELISA kit were included in this study and were designated as P1–P85. The HspA antibody could be detected in 42 of 85 persons by ELISA, and the positivity rate was 49.4%. Among the 42 HspA seropositive persons, 9 were seropositive for HP11 epitope and 14 were seropositive for HP19 epitope, with positive rates of 21.4% and 33.3%, respectively. Control sera from the 17 *H. pylori* IgG-negative humans (N1–N17) did not respond to epitopes and HspA. We mapped the antibody profile of two epitopes in HspA seropositive persons (Figure 5).

## 4. Discussion

HspA is a GroES-like protein with a calculated molecular mass of 13 kDa, which, as expected, is present in all *H. pylori* strains [8]. HspA consists of two distinct domains, which suggest that it may be a bifunctional protein involved in both of the classical GroES chaperoning functions (A domain) as well as a nickel-carrier function, mediated by the B domain [17]. HspA seropositivity in *H. pylori*-positive subjects ranges from 39 to 60% [13,18,19,20], which correlates significantly with age. In this study, HspA antibody could be detected in 42 of 85 persons with *H. pylori* infection by ELISA with a positive rate of 49.4%, which was consistent with previous findings. Previous research showed that the dominant serological response in the subjects was directed against the A domain of HspA, while the B domain of HspA appeared to be weakly immunoreactive [17]. The two B-cell epitopes identified in this work are both located in the A domain, demonstrating that the immunodominant segment of HspA is located in the A domain.

First, we screened and identified antigen epitopes in HspA using polyclonal antibodies obtained from HspA whole protein immunization. After two truncations, we finally located two 11-amino acid peptides, HP11 and HP19, which may be epitopes of HspA. Next, we demonstrated that the epitopes HP11 and HP19 had good immunogenicity. As the epitope was too short to directly stimulate the body to produce an immune response, we conjugated the epitope peptide to KLH and injected it into immunize mice to test whether they could produce peptide-specific antibodies. As expected, the conjugated peptides could produce epitope-specific antibodies when injected at a high titer. Epitope specific anti-sera can recognize the naive HspA, rHspA, and self-GST fusion peptides. Furthermore, using rHspA anti-serum to recognize the GST fusion peptide of the two epitopes, the corresponding immune response was also observed. These results confirmed that HP11 and HP19 had good antigenicity, and the anti-rHspA polyclonal antibody contained anti-HP11 and anti-HP19 antibodies. When the anti-sera reacted with the naive HspA and rHspA in Western blots, two or three bands could be seen, which corresponded to monomeric, dimeric, and trimeric forms of HspA. This was also consistent with the literature [21]. When the epitope peptide stimulated the mouse spleen lymphocytes, a clear lymphocyte proliferation response could be seen. This indicated that the epitope peptide could be recognized by spleen lymphocytes in immunized mice and stimulated the proliferation of splenic lymphocytes.

HP11 and HP19 are two antigenic epitope peptides with high sequence conservation. Identifying conserved pathogenic antigens or epitopes that can stimulate the host’s immune response is an important way of studying pathogenic mechanisms and obtaining highly efficient recombinant vaccines [22,23]. Serum samples of 85 *H. pylori* IgG-positive and 17 *H. pylori* IgG-negative humans were enrolled in this study. In HspA seropositive persons, the seropositivity rates of HP11 and HP19 were 21.4% and 33.3%, respectively. This means that HP11 and HP19 are immunodominant epitopes and have a significant antibody positive rate in naturally infected persons. These two epitopes provided new candidate molecules for molecular vaccine design and diagnostic reagent development of *H. pylori* based on a multi epitope combination strategy.

## 5. Conclusions

In summary, we successfully identified two B-cell epitopes of HspA, both of which were located in the A domain. These two epitopes had good antigenicity and immunogenicity, as well as a high degree of conservation. The positive rates of epitope antibodies in HspA seropositive persons infected with *H. pylori* were 21.4% and 33.3%, respectively. These new B-cell epitopes of HspA have potential value for studying the immune response of *H. pylori* infection and for designing an epitope-based vaccine against *H. pylori*.

## 6. Patents

The patents related to this research have been applied for.

## Data Availability

Not applicable.

## Supplementary Materials

The following supporting information can be downloaded at: https://www.mdpi.com/article/10.3390/vaccines10010065/s1, Table S1: Conservative analysis of amino acid sequence of HP11 epitope peptide; Table S2: Conservative analysis of amino acid sequence of HP19 epitope peptide.
